# Survey data on teaching strategies and product innovation: A focus on selected university students in Nigeria

**DOI:** 10.1016/j.dib.2018.03.027

**Published:** 2018-03-12

**Authors:** Maxwell Olokundun, Stephen ibidunni, Mercy Ogbari, Fred Peter, Taiye Borishade, Hezekiah Falola, Odunayo Salau, Oladele Kehinde

**Affiliations:** Covenant University, Nigeria

**Keywords:** Teaching strategies, Product innovation, University students

## Abstract

The main objective of this study was to examine the effectiveness of the teaching methods adopted in motivating university students’ in Nigeria to engage in product innovation. Emphasis was laid on Covenant University in Nigeria which is the pioneer institution to offer entrepreneurship education in Nigeria. The study adopted quantitative method with a descriptive research design to establish trends related to the objective of the study. Survey was be used as quantitative research method. The population of this study comprised all students in the selected university which was given as 6401 3. A sample size of 377 students was selected using yard's formula. Reliability and validity were confirmed. Data was analyzed with the use of Statistical Package for Social Sciences (SPSS).Regression analysis was used as statistical tool of analysis. The field data set is made publicly available to enable critical or a more extensive inquiry.

**Specification table**

**Table:** 

**Subject area**	Business, Management
**More Specific Subject Area:**	Business and Entrepreneurship education
**Type of Data**	Table
**How Data was Acquired**	Researcher-made questionnaire analysis
**Data format**	Raw, analyzed, Descriptive and Inferential statistical data
**Experimental Factors**	Sample consisted of university students in Nigeria. The researcher-made questionnaire which contained data on teaching methods and product innovation were completed..
**Experimental features**	Teaching methods are one of the factors endangering entrepreneurial development of university students.
**Data source location**	South west Nigeria
**Data Accessibility**	Data is included in this article

**Value of data**

These data present data on teaching methods in university entrepreneurship education as it relates to creating salient entrepreneurial experience for undergraduates. This is geared towards development of relevant entrepreneurial proficiencies by university students.

The results showed that the use of experiential teaching methods can be very helpful for universities and entrepreneurship educators in achieving desired results for university entrepreneurship education.

The results of this study can be used to improve pedagogical practices in university entrepreneurship education.

## 1 Data

As shown in Table 1 below, the research questionnaire was administered to three hundred and seventy-seven (377) respondents representing the sample size used in the university selected (Covenant University). Three hundred (300) copies were returned, and seventy-seven (77) copies were not returned.

**Table 1:** Analysis of general response rate.

**Questionnaire**	**Respondents**	**Percentage of respondents**
Retrieved	300	79.6%
Not retrieved	77	20.4%
Total	377	100%

Based on the copies of questionnaire retrieved, the following represents the personal (Bio) data section of questionnaire. It shows a comprehensive table indicating the gender, age, educational attainment, self- employment and level of the respondents.

The above Gender Distribution shows that 135(45.0%) persons are male respondents while 165(55.0%) are female respondents. The female respondents have the highest percentage, which implies that majority of the research questionnaire were filled by females.

The above table shows that 57(19.0%) of the respondents were within the range of (16-19) years of age, 227 (75.7%) of the respondents were within the age of (20-23), and 16(5.3%) of the respondents were within the age of (24-27). This implies that the majority of the respondents were within the age of (20-23).

The above table shows that 300(100%) of the respondents have WASSCE/ O LEVEL as their highest level of educational attainment.

The above table shows that 128(42.7%) of the respondents are self-employed while 172(57.3%) are not self- employed. This implies that the majority of the respondents are not self-employed.

This table above shows that 48(16.0%) of the respondents are in 200 level while 95(31.7%) of the respondents are in 300 level, 111(37.0%) of the respondents are in 400level, and 46(15.3%) of the respondents are in 500 level. This implies that the majority of respondents are in 400 level.

From the above table, responses to the research statement “The teaching and learning strategies used in this course is of high standard” are interpreted thus; (0.3%) of respondents strongly disagree, (16.0%) disagree, (14.7%) undecided, (45.7%) agree and (23.3%) strongly agree.

From the above table, responses to the research statement “There are different teaching methods used in this course” are interpreted thus; (12.3%) of respondents disagree, (15.7%) undecided, (48.3%) agree and (23.7%) strongly agree.

From the above table, responses to the research statement “The Teaching methods used in this course have influenced my mindset concerning product innovation” are interpreted thus; (0.3%) of respondents strongly disagree, (6.0%) disagree, (9.0%) undecided, (65.3%) agree, (19.3%) strongly agree.

From the above table, responses to the research statement “I have developed products that are of high value and can be easily differentiated from the existing ones” are interpreted thus; (2.7%) of respondents disagree, (10.3%) undecided, (63.0%) agree and (24.0%) strongly agree (Table 2, Table 3, Table 4, Table 5, Table 6, Table 7, Table 8, Table 9, Table 10).

**Table 2:** Percentage distribution of gender of the students.

		Frequency	Percent	Valid percent	Cumulative percent
Valid	Male	135	45.0	45.0	45.0
	Female	165	55.0	55.0	100.0
	Total	300	100.0	100.0

**Table 3:** Age distribution of the respondents.

		Frequency	Percent	Valid percent	Cumulative percent
Valid	16–19	57	19.0	19.0	19.0
	20-23	227	75.7	75.7	94.7
	24-27	16	5.3	5.3	100.0
	Total	300	100.0	100.0

**Table 4:** Educational attainment of respondents.

		Frequency	Percent	Valid percent	Cumulative percent
Valid	WASSCE/ O LEVEL	300	100.0	100.0	100.0

**Table 5:** Self- employment of respondents.

		Frequency	Percent	Valid percent	Cumulative percent
Valid	Yes	128	42.7	42.7	42.7
	No	172	57.3	57.3	100.0
	Total	300	100.0	100.0

**Table 6:** Level of respondents.

		Frequency	Percent	Valid percent	Cumulative percent
Valid	200	48	16.0	16.0	16.0
	300	95	31.7	31.7	47.7
	400	111	37.0	37.0	84.7
	500	46	15.3	15.3	100.0
	Total	300	100.0	100.0

**Table 7:** Descriptive statistics measuring the standard of teaching methods used in the course.

		Frequency	Percent	Valid percent	Cumulative percent
Valid	Strongly disagree	1	.3	.3	.3
	Disagree	48	16.0	16.0	16.3
	Undecided	44	14.7	14.7	31.0
	Agree	137	45.7	45.7	76.7
	strongly agree	70	23.3	23.3	100.0
	Total	300	100.0	100.0

**Table 8:** Descriptive statistics measuring variations in teaching methods used.

		Frequency	Percent	Valid percent	Cumulative percent
Valid	Disagree	37	12.3	12.3	12.3
	Undecided	47	15.7	15.7	28.0
	Agree	145	48.3	48.3	76.3
	Strongly agree	71	23.7	23.7	100.0
	Total	300	100.0	100.0

**Table 9:** Descriptive statistics measuring teaching methods and the development of innovative mindset.

		Frequency	Percent	Valid percent	Cumulative percent
Valid	Strongly disagree	1	.3	.3	.3
	Disagree	18	6.0	6.0	6.3
	Undecided	27	9.0	9.0	15.3
	Agree	196	65.3	65.3	80.7
	strongly agree	58	19.3	19.3	100.0
	Total	300	100.0	100.0

**Table 10:** Descriptive statistics measuring teaching methods and product innovation.

		Frequency	Percent	Valid percent	Cumulative percent
Valid	Disagree	8	2.7	2.7	2.7
	Undecided	31	10.3	10.3	13.0
	Agree	189	63.0	63.0	76.0
	Strongly agree	72	24.0	24.0	100.0
	Total	300	100.0	100.0	

Based on the inferential statistical data, the test statistics used in this hypothesis is the regression analysis. The adoption of this test statistics is based on the fact that regression analysis is used in describing the dependence of variable on one or more variables. It is also used to determine the effect and the impact of the independent variable on the dependent variable.

The formula for multiple regression is Y’= = α + β_1_X_j_ + β_2_×_2j_ + ……….β_k_X_j_.

WHERE Y’ = the dependent variable

α = the value of the intercept
β = the slope of the Independent Variable
X = the Independent variable

The F ratio is calculated to determine the level of significance and at an appropriate degree of freedom, R square is the coefficient of determination, the strength and direction of the relationship. Beta determines the relative importance of the independent variable. Standard errors display the strength of standard deviation, the higher the standard error, the less significance and the lower the standard error, the greater the significance. The significance level below 0.05 implies a statistical confidence of above 95%. Therefore, we reject the null hypothesis once the P- value is < 0.05 and accept the alternative hypothesis

H_0_: There is no significant effect of teaching methods on product innovation.

Regression analysis was used in evaluating the Hypothesis. Table 11 represents the ‘Model Summary’ and it describes to what extent the dependent variable is explained by the model. Therefore, the outcome for the table shows that teaching methods has an effect of 42.3%. i.e. (R square = (0.423*100)) on product innovation.

**Table 11:** Model summary.

Model	R	R square	Adjusted R square	Standard error of the estimate
1	.650^a^	.423	.421	.54320

a Predictors: (Constant), product innovation

The Analysis of Variance table tests the null hypothesis to determine if it is statistically significant. From the results, the model in Table 12 is statistical significance (F (1.298) = 218.572, p = .000). The statistical significance in the above table is (.000), therefore the null hypothesis should be rejected because the P – value is less than 0.05 significant level. This implies that teaching methods has a significant effect on product innovation’

**Table 12:** Analysis of variance^a^.

Model		Sum of squares	Degree of freedom	Mean square	F	Significance
1	Regression	64.493	1	64.493	218.572	.000^b^
	Residual	87.929	298	.295		
	Total	152.422	299			

a Dependent Variable: product innovation

b Predictors: (Constant), teaching methods

Coefficient Table 13 shows the simple model that expresses the effect of teaching methods on product innovation. In this table, the beta co-efficient is 0.650, which relates to product innovation. From this table, we can conclude that teaching methods has significant effect on product innovation as (14.784) is greater than slope 0.882 and sig = (0.000). The hypothesis generated a level of significance of 0.000 which is less than 0.05 (p < 0.05) which is the standard for rejecting the null hypothesis and accepting alternate hypothesis. This aligns with the study of [7] who opined that the development of entrepreneurial competencies by university students is hinged on instructional strategies. This also corresponds with the work of [1] which showed that the appropriate teaching methods for the development of entrepreneurial competencies by university students

**Table 13:** Coefficient^a^.

Model		Unstandardized coefficients		Standardized coefficients	t	Significance
		B	Standard. error	Beta		
1	(Constant)	.192	.250		.770	.442
	product innovation	.882	.060	.650	14.784	.000

a Dependent Variable: product innovation

## 2 Experimental design, materials and methods

Covenant University was selected from South west Nigeria. Three hundred and seventy seven students were selected to participate in this study. Data were gathered from students across the various colleges in the selected university with the aid of a researcher- made questionnaire based on the works of [2], [3], [4], [5], [6], [8], [9]. The collected data were coded and entered into SPSS version 22 Data analysis was performed; using SPSS-22 Data was analyzed applying inferential statistical tests which involved regression analysis. There was a meaningful relationship between teaching methods and engagement in product innovation in the selected university in south west Nigeria.

## 3 Conclusion and implications for the study

This study revealed that teaching methods have significant and positive impact on students’ product innovation. The requisite for university students to develop entrepreneurial competences and proficiencies while in school obliges universities to appreciate the importance of engaging appropriate teaching methods particularly with regards to the propensity of students to engage in product innovation hence, this present study has extensive implications for both the universities, entrepreneurship educators and undergraduate students in this regard. To this end, the data presented in this article is imperative for more comprehensive analysis or investigation.
